# Molecular-guided therapy for the treatment of patients with relapsed and refractory childhood cancers: a Beat Childhood Cancer Research Consortium trial

**DOI:** 10.1186/s13073-024-01297-5

**Published:** 2024-02-12

**Authors:** Giselle L. Saulnier Sholler, Genevieve Bergendahl, Elizabeth C. Lewis, Jacqueline Kraveka, William Ferguson, Abhinav B. Nagulapally, Karl Dykema, Valerie I. Brown, Michael S. Isakoff, Joseph Junewick, Deanna Mitchell, Jawhar Rawwas, William Roberts, Don Eslin, Javier Oesterheld, Randal K. Wada, Devang Pastakia, Virginia Harrod, Kevin Ginn, Raya Saab, Kevin Bielamowicz, Jason Glover, Eugenia Chang, Gina K. Hanna, Daniel Enriquez, Tyler Izatt, Rebecca F. Halperin, Abigail Moore, Sara A. Byron, William P. D. Hendricks, Jeffrey M. Trent

**Affiliations:** 1 Division of Pediatric Hematology/Oncology, Penn State Health Children’s Hospital, 500 University Drive, MC-H085, Rm. C7621, Hershey, PA 17033-0850 USA; 2 Levine Children’s Hospital, Atrium Health, Charlotte, NC USA; 3 Medical University of South Carolina, Charleston, SC USA; 4 Cardinal Glennon Children’s Medical Center, St. Louis University School of Medicine, St. Louis, MO USA; 5 Connecticut Children’s Medical Center, Hartford, CT USA; 6 Helen DeVos Children’s Hospital, Spectrum Health, Grand Rapids, MI USA; 7 Children’s Hospitals and Clinics of Minnesota, Minneapolis, USA; 8 Rady Children’s Hospital-San Diego and UC San Diego School of Medicine, San Diego, CA USA; 9 St. Joseph’s Children’s Hospital, Tampa, FL USA; 10 Kapiolani Medical Center for Women and Children, University of Hawaii, Honolulu, HI USA; 11 Vanderbilt-Ingram Cancer Center, Nashville, TN USA; 12 Dell Children’s Blood and Cancer Center, Ascension Dell Children’s, Austin, TX USA; 13 Children’s Mercy, Kansas City, MO USA; 14 Stanford Medicine Children’s Health, Palo Alto, CA USA; 15 Arkansas Children’s Hospital, Little Rock, AR USA; 16 Randall Children’s Hospital, Portland, OR USA; 17 St. Luke’s Cancer Institute, Boise, ID USA; 18 Orlando Health Cancer Institute, Orlando, FL USA; 19 Translational Genomics Research Institute, Phoenix, AZ USA

**Keywords:** Neuroblastoma, CNS tumors, Rare tumors, Orphan diseases, Molecular-guided therapy, Pediatric oncology, Genomic sequencing

## Abstract

**Background:**

Children with relapsed central nervous system (CNS tumors), neuroblastoma, sarcomas, and other rare solid tumors face poor outcomes. This prospective clinical trial examined the feasibility of combining genomic and transcriptomic profiling of tumor samples with a molecular tumor board (MTB) approach to make real‑time treatment decisions for children with relapsed/refractory solid tumors.

**Methods:**

Subjects were divided into three strata: stratum 1—relapsed/refractory neuroblastoma; stratum 2—relapsed/refractory CNS tumors; and stratum 3—relapsed/refractory rare solid tumors. Tumor samples were sent for tumor/normal whole-exome (WES) and tumor whole-transcriptome (WTS) sequencing, and the genomic data were used in a multi-institutional MTB to make real‑time treatment decisions. The MTB recommended plan allowed for a combination of up to 4 agents. Feasibility was measured by time to completion of genomic sequencing, MTB review and initiation of treatment. Response was assessed after every two cycles using Response Evaluation Criteria in Solid Tumors (RECIST). Patient clinical benefit was calculated by the sum of the CR, PR, SD, and NED subjects divided by the sum of complete response (CR), partial response (PR), stable disease (SD), no evidence of disease (NED), and progressive disease (PD) subjects. Grade 3 and higher related and unexpected adverse events (AEs) were tabulated for safety evaluation.

**Results:**

A total of 186 eligible patients were enrolled with 144 evaluable for safety and 124 evaluable for response. The average number of days from biopsy to initiation of the MTB-recommended combination therapy was 38 days. Patient benefit was exhibited in 65% of all subjects, 67% of neuroblastoma subjects, 73% of CNS tumor subjects, and 60% of rare tumor subjects. There was little associated toxicity above that expected for the MGT drugs used during this trial, suggestive of the safety of utilizing this method of selecting combination targeted therapy.

**Conclusions:**

This trial demonstrated the feasibility, safety, and efficacy of a comprehensive sequencing model to guide personalized therapy for patients with any relapsed/refractory solid malignancy. Personalized therapy was well tolerated, and the clinical benefit rate of 65% in these heavily pretreated populations suggests that this treatment strategy could be an effective option for relapsed and refractory pediatric cancers.

**Trial registration:**

ClinicalTrials.gov, NCT02162732. Prospectively registered on June 11, 2014.

**Supplementary Information:**

The online version contains supplementary material available at 10.1186/s13073-024-01297-5.

## Background

Childhood cancer is the second leading cause of death in children ages 1–14 and the fourth most common cause of death in adolescents ages 15–19 [1]. Although improvements in the past 40 years have led to markedly improved survival rates approaching 80% for pediatric cancers in general, patients with relapsed and advanced stage tumors continue to have very poor prognoses with overall survival rates below 20% [2]. Of these, relapsed CNS tumors, neuroblastoma, sarcomas, and other rare solid tumors pose the greatest challenge.

Cancer results from disruption of molecular pathways within cells due to genetic or epigenetic events or changes in the tumor [3–5]. The molecular networks engaged during tumor development and progression are complex and constantly evolving to provide resistance against normal protective measures, allowing cells to adapt to or exploit extracellular cues [6]. This complexity is further exacerbated by the genomic instability seen in many cancer cells, which leads to an accelerated evolutionary process that results in subclones and further heterogeneity [7, 8]. This variability, combined with the adaptability of many molecular pathways, provides a path to resistance against agents that target a subset of cellular systems within a tumor’s molecular and genetic repertoire [9].

A fundamental challenge in targeted cancer treatment is identifying optimal therapeutic combinations that can treat heterogeneous tumors that are both highly adaptive and that exhibit significant inter‑ and intra‑patient variation [3, 10–12]. The growth of next-generation sequencing and targeted gene panels has led to a shift in the oncology world from a histology-driven approach to a tumor-agnostic molecularly driven approach. This has been especially beneficial for common adult cancers with high mutation burdens and known genetic markers that can easily be identified on limited panels. However, the utility of gene panels is limited in the world of rare pediatric cancers, given their paucity of actionable mutations. Unlike adult cancers which are often driven by single nucleotide variant (SNV) mutations, pediatric cancers are more frequently driven by structural variants and epigenetic perturbations, resulting in an aberrant RNA expression profile. A holistic approach including whole-exome sequencing, RNA expression profiling, and epigenetic analysis is needed to identify molecular disruptions among pediatric cancers [13].

With the expansion of genomic profiling comes the advent and increasing clinical utility of small molecule inhibitors. The hypothesis that genomic alterations can be identified via genomic sequencing and can be matched with specific inhibitors that target pathways resulting in effective targeted therapy and reduced toxicities is the basis of precision medicine. Several of these targeted agents have shown efficacy in pediatric cancers, as in the use of ALK inhibitors such as crizotinib in ALK-mutated neuroblastoma (NB) [14] and CDK4/6 inhibitors including Palbociclib in pediatric sarcomas [15].

Pediatric precision medicine trials address the diversity in cancers by undertaking a histology-agnostic approach. A limitation of such trials has been the use of single-agent therapy when actionable alterations were identified, limiting the therapeutic benefit to patients and increasing the chances of tumor resistance. Studies with single agents alone are less likely to produce clinical benefit (28%) when compared to multi-agent trials (71%) [16]. From previous studies, the feasibility of comprehensive profiling has been demonstrated [17–26]. Here, we integrate comprehensive sequencing and multi-agent treatment as we assess the safety, feasibility, and efficacy of a precision medicine approach to treat all high‑risk childhood solid malignancies. This clinical trial includes a variety of pediatric solid tumors that underwent comprehensive genomic analysis, including whole-exome sequencing and transcriptome analysis, and a molecular tumor board wherein all subjects received an individualized multi-agent treatment plan in a novel “*N* of one” study design. We hypothesize that genomic sequencing and molecular tumor boards will lead to clinical responses in patients who otherwise lack standard treatment options.

## Methods

### Study design

This was a prospective, open‑label, multicenter study. Subjects were enrolled in the Beat Childhood Cancer (BCC), formerly Neuroblastoma Medulloblastoma Translational Research Consortium (NMTRC), clinical trial NMTRC009. This study was prospectively registered on ClinicalTrials.gov on June 11, 2014, with identifier: NCT02162732, and enrolled from July 8, 2014, to June 10, 2018. This study was approved by the Western Institutional Review Board (IRB) in addition to all local IRBs at the 17 enrolling institutions. Informed consent was obtained from all subjects enrolled in this study. This study was conducted according to the principles of the 2004 version of the Declaration of Helsinki, the International Conference on Harmonization Guidance on Good Clinical Practice (ICH GCP), and the requirements of all local regulatory authorities regarding the conduct of clinical trials and the protection of human subjects. Study data were collected and managed using REDCap electronic data capture tools hosted at BCC [27, 28]. This study is now completed and is no longer enrolling.

The primary objective was to determine the feasibility of combining genomic profiling of tumor samples with a drug-gene matching algorithm to make real‑time treatment decisions for children with relapsed/refractory solid tumors. Feasibility was assessed at the end of cycle 1 using the following feasibility definition: enrollment into study, genomic profile, analysis and report generation completed, tumor board held with treatment decision, treatment review completed, start of treatment, and completion of 1 cycle of therapy.

The secondary objective was to determine the overall response rate (ORR) by the presence of radiologically assessable disease by cross-sectional CT or MRI imaging and/or by MIBG or PET scans. The assessment of response included the initial measurable targets, and then an assessment was performed after cycle 2 and then after every two additional cycles. An additional secondary objective was the safety analysis that was to be conducted on all subjects who have received at least one dose of therapy and included the frequency of all reported adverse events and laboratory abnormalities as well as the frequency of dose interruptions, dose reductions, and treatment discontinuation. Adverse events were collected from the day of the first dose of MGT therapy until 30 days after the last dose.

In addition, tumor tissue was collected for correlative biologic studies. Tumor cells were grown to 70% confluency in cell growth media and established as cell lines. Upon successful cell line generation, tumor cells were then implanted into non-obese, diabetic severe combined immunodeficient (NOD‑SCID) mice for patient-derived xenograft (PDX) creation.

### Subjects enrollment criteria

Eligible subjects were required to have a confirmed diagnosis of pediatric cancer fitting into one of three general diagnostic categories (neuroblastoma, CNS tumor, or other rare solid tumors) that was either refractory to established proven therapies or for which there was no known effective curative therapy. Subjects were required to be ≤ 21 years of age at initial diagnosis, > 12 months of age at enrollment, have a Lansky/Karnofsky performance score ≥ 50, and have measurable disease as defined by the Response Evaluation Criteria in Solid Tumor version 1.1 (RECIST v1.1) [29] with at least one tumor lesion accessible for biopsy. Screening of subjects was performed within 14–21 days prior to biopsy. Written, informed consent was obtained according to institutional guidelines. Tumor samples submitted for analysis had to be > 50% viable, confirmed by local pathology. Subjects with disease confined to the bone marrow were also eligible to enroll if the degree of marrow involvement was > 50%. Subjects who were expected to have no evidence of disease (NED) after surgical removal of their tumor were still eligible for this trial if their disease typically required adjuvant chemotherapy treatment after surgery, despite NED status. Specimens were obtained only in a non‑significant risk manner and not solely for investigational testing.

**Fig. 1:**
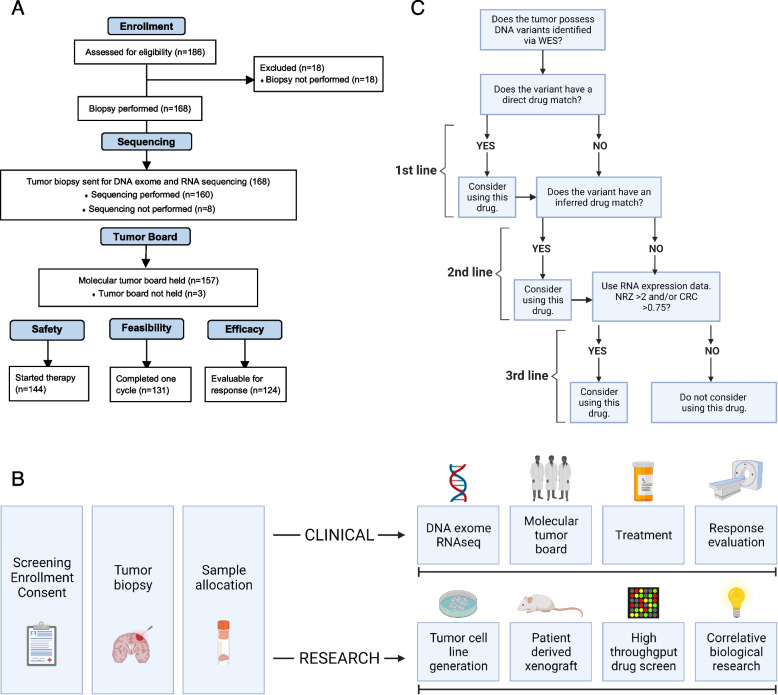
**A** The consort diagram includes the flow of inclusion from enrollment through safety, feasibility, and efficacy criteria. A total of 186 patients were consented for this clinical trial. A total of 144 started MGT, 131 completed one full cycle of MGT, and 124 were evaluable for best response. **B** The study flow diagram includes the flow of data and samples throughout the trial. Upon consent and enrollment on‑study, patients underwent biopsies to obtain tumor samples in a non‑significant fashion. Tumor samples were then divided between the clinical and research arms and sent to Ashion (CLIA) and POTRL (non‑CLIA) for further evaluation. Tumor-normal whole-exome sequencing (WES) and tumor RNA sequencing were performed at Ashion. Data were analyzed, and the results were used to guide treatment decisions through a molecular tumor board. Patients received therapy according to the molecular tumor board and were evaluated for response per protocol. Tumor samples sent to POTRL were used for cell line and PDX model generation, high-throughput drug screening, and further biological research and analysis. **C** The drug algorithm represents the flow of decision-making that occurs during the molecular tumor board in order to devise the combination of MGT. Direct or inferred drug matches were first- and second-line options, respectively. However, due to the historically low mutational burden of pediatric tumors, RNA data was primarily used for drug selection. The algorithm was cycled up to four times until the combination therapy treatment plan was decided. *Repeat the algorithm until a three- or four-drug combination has been decided

There were 186 subjects screened for this study. Of these, 18 were excluded due to the inability to obtain a biopsy. Of the 168 who had biopsies performed, 160 of the tumor samples completed tumor-normal whole-exome sequencing and tumor RNA sequencing. Molecular tumor boards were held for 157 of the subjects who had adequate sequencing. Of those, 144 of these subjects started molecular‑guided therapy based on the tumor board recommendations and were evaluable for safety. A total of 131 subjects met the feasibility criteria, having completed at least 1 cycle of treatment, and 124 subjects had at least 1 set of scans following completion of at least 1 cycle of therapy and were evaluable for efficacy. See consort diagram for more information (Fig. 1A).

### Endpoints

The primary endpoint of this clinical trial was the feasibility of sequencing biopsied tumor samples, generating a genomic profile, allowing a multidisciplinary tumor board to devise a molecular-guided personalized treatment plan, initiating combination therapy, and completing 1 cycle of therapy. This endpoint was measured in days from biopsy to each time point. The secondary endpoints of this study were to (a) evaluate the safety of combining up to four targeted agents as measured by the number of subjects with related and unexpected adverse events, medication holds, discontinued medications, and dose reductions during the first two cycles of molecular guided therapy; (b) evaluate the efficacy of the treatments chosen using best response while on-study; and (c) explore the relationship between tumor phenotype/genotype and response.

### Sample collection

There were 168 subjects who underwent a scheduled surgical resection and/or diagnostic biopsy procedure in which fresh tumor samples and a blood sample were collected. The fresh tumor samples were flash-frozen in dry ice and shipped to Ashion Analytics (http://www.ashion.com), a CLIA‑certified laboratory, for tumor-normal whole-exome sequencing and tumor RNA sequencing. Blood samples were shipped at room temperature to Ashion Analytics and underwent DNA germline sequencing. A sample of fresh tumor was collected using a sterile technique and placed within 20 min in a T25 flask containing cell growth media, sealed with parafilm, and shipped overnight at ambient temperature to the research laboratory (Fig. 1B).

### Somatic variant analysis

Ashion’s Strexome assay, which included tumor-normal whole-exome and tumor mRNA sequencing (RNASeq), was performed on the Illumina HiSeq 2500 Sequencer with alignment to build 37 of the human reference genome, as previously described [30, 31]. Variant calls from Ashion were used in the variant-drug matching algorithm for discussion at the molecular tumor boards. Briefly, Seurat [32] was used for calling somatic single nucleotide variants and small indels, a custom copy number tool (https://github.com/tgen/tCoNuT) was used to call focal copy number events, Manta (https://github.com/Illumina/manta) was used for structural variant calling, and TopHat fusion (v2.0.8b, RRID:SCR_013035) was used for fusion detection. In follow-on research analysis, ExomeCNV (RRID:SCR_010815) was used to call chromosome arm-level events, filtering for regions containing 50 or more genes with a log2 ratio of tumor to matched normal of ≥ 0.5 (gain) or ≤  − 0.5 (loss). Chromosome arm-level events were called if the gain or loss covered ≥ 50% of the arm. Events seen in ≥ 15 tumors are included in the oncoprint. Quality control (QC) thresholds included metrics of base calling quality, coverage, allelic read percentages, strand bias, and alignment quality. Somatic tumor mutation burden was evaluated using an in-house tool to calculate the number of somatic point mutations per megabase (Mb).

### Gene expression analysis

For gene expression analysis, sequence read processing included read trimming with Trimmomatic-0.36 [33], alignment with STAR 2.5.3 to GRCh37 [34], read counts using R package GenomicAlignments [35], and regularized logarithm (rlog) values using DESeq2 [36]. A previously published standardized *Z*‑score method was used to suggest anomalous expression in each sample based on comparison with a whole‑body reference of 22 normal tissue gene expression levels [18]. This method known as the normal reference using a standard *Z*‑score (NRZ), assigned a *Z*‑score to each gene depending on the number of standard deviations they were from the normal sample population mean, either over- or underexpression of certain biomarkers. In addition, tumor gene expression profiles for each participant’s tumor were compared to the expression profiles of other childhood relapsed/refractory tumors (66 samples from 54 subjects with neuroblastoma, CNS tumors, or sarcoma), known as the cancer reference, based on the cumulative distribution-based statistic (CRC). A heatmap was generated from NRZ scores using the Complex Heatmap R package [37].

### Mutational signature analysis

Mutational signature analysis was performed for tumor samples using YAPSA [38] with COSMIC signatures v2 [39] under default parameters. The proportion of mutations contributing to each signature was calculated within a sample and then averaged within tumor types [38, 40].

### Longitudinal analysis

Genomic evolution was evaluated for subjects with multiple longitudinal samples sequenced on the study. In comparing the sequencing results from biopsies at each distinct time point, a phylogeny of mutations was established, outlining the evolution and subsequent heterogeneity of tumors. Nine subjects had two longitudinal biopsies; three subjects had three longitudinal biopsies. Venn diagrams were generated using an in-house script to show protein-coding mutations, CNVs, and fusions that were shared and unique across three longitudinal biopsies in the three subjects. A heatmap was generated from NRZ scores of drug-targetable genes using the Complex Heatmap R package [37]. Inkscape graphics editor was used to render shapes, colors, and text (RRID:SCR_014479).

### Drug prediction report

The encrypted de-identified sequencing data was processed and securely uploaded for the creation of the Drug Prediction Report. The drug matching algorithm utilized in determining the molecular‑guided treatment plan contained three tiers of decision-making. The first tier was a direct variant/drug matching in which there was literature evidence indicating that the variant had been directly associated with a change in response to therapy, or the gene was a target of the drug. The second tier was an inferred variant/drug matching in which the literature indicated that the variant was in a gene that is a direct target of a drug that was computationally predicted to have a biological impact on the gene. The third tier was based on the RNA expression of biomarkers and drug targets. In this methodology, read counts were converted to a relative measure of transcript abundance, and NRZ was used to determine statistically significant differences in each sample compared to normal tissues, as previously described [18, 41]. The BCCLIMS contained a database of drugs and genes with a column that implied whether over‑ or underexpression of that gene, calculated by the NRZ, indicated potential sensitivity or resistance to the drug, as well as literature-based evidence supporting that rule.

### Treatment

Treatment protocols were generated from the molecular tumor board meeting after discussion of the medical summary, the information contained in the report generated from genomic DNA exome and RNA transcriptome analysis of the subject’s tumor, and literature-based evidence. The molecular tumor board consisted of pediatric oncologists, pharmacists, genomics experts, cancer biologists, and bioinformaticians. Three reviewers were assigned to each case, presenting overview and literature evidence to the clinical team, who then voted to approve recommended therapies. All agents were FDA-approved drugs with published age-appropriate dosing which was reviewed by the pharmacist. Potential drug choices were analyzed regarding safety, mechanism, availability, and cost. Drug combinations were allowed, up to a maximum of four agents. Previously established and tested regimens were given priority. A pharmacist analyzed the potential drug interactions between the targeted agents and the patient’s routine medications and supplements. The final treatment regimen was subjected to an in‑depth review and evaluation of safety and signed off by a study pharmacist. A treatment memo outlining the regimen, known and potential adverse events, and any additional recommended clinical monitoring was reviewed with and signed by the subject or subject’s legal representative.

### Assessments

Response was determined every two cycles via CT/MRI, MIBG/PET scans, and/or bone marrow assessment and was classified using RECIST v1.1 [29]. Patients with evidence of bone marrow disease were evaluated in terms of disease presence in bone marrow aspirates/biopsies. Disease evaluation scans were sent to the BCC for central review.

All adverse events were described in the source documents and graded according to the Common Terminology Criteria for Adverse Events (CTCAE) v4.0 [42]. Grade 3 or higher related and unexpected adverse events that occurred during the study were captured. To further assess the feasibility and safety of initiating a three- to four-drug combination of MGT, a review of treatment roadmaps was performed, capturing the date of initiation of combination therapy, total number of cycles started, and toxicity-associated events that occurred during cycles 1 and 2 (medication holds, dose modifications, medication discontinuations, and cycle delays).

## Results

### Patient characteristics

Subjects were enrolled from July 8, 2014, to June 10, 2018. Table 1 describes the characteristics of all subjects who met the safety criteria (*n* = 144). The average age of subjects at study enrollment was 11.04 years old, with 56.94% ≥ 10 years old. There were three subjects (all with diffuse midline glioma, H3.3K27M‑mutant) who were new diagnoses and therefore did not receive any chemotherapy, radiation, or surgery prior to enrollment, given the lack of curative therapy for their disease state. Over half of all subjects had received at least one line of relapse therapy prior to initiation of the recommended molecular‑guided therapy regimen (69.44%). The average amount of time from the initial diagnosis to study enrollment was 3.10 years with a range of 0 to 19 years. 21.53% of the subjects (31/144) had neuroblastoma. CNS tumors comprised 28.47% of the subjects (41/144), with ependymoma being the most common (9/144, 6.25%). Rare tumors comprised 50.00% of the subjects (72/144), with rhabdomyosarcoma being the most common (17/144, 11.81%).

**Table 1:** Patient characteristics were evaluated using the sample size of all subjects meeting safety criteria, *n* = 144

NMTRC009 patient characteristics	*N* = 144
**Enrollment age, years**	
Mean (range)	11.04 (1, 23)
Median	11
≥ 10, *n* (%)	82 (56.94%)
< 10, *n* (%)	62 (43.06%)
**Sex, *n* (%)**	
Female	62 (43.06%)
Male	82 (56.94%)
**Race/ethnicity, *n* (%)**	
White	93 (64.58%)
Black/African American	10 (6.94%)
Asian	5 (3.47%)
Hispanic	13 (9.03%)
Others/unknown	23 (15.97%)
**Lansky status, *n* (%)**	
≥ 80	125 (86.81%)
< 80	19 (13.19%)
**Previous relapse treatment lines, *n* (%)**	
New diagnosis	3 (2.08%)
0	41 (28.47%)
1	36 (25.00%)
2	19 (13.19%)
≥ 3	45 (31.25%)
**Time from diagnosis to enrollment, years**	
Mean (range)	3.1 (0, 19)
Median	2
**Tumor type, *n* (%)**	
**Neuroblastoma**	31 (21.53%)
**CNS tumor**	41 (28.47%)
Ependymoma	9 (6.25%)
Glioblastoma	8 (5.55%)
Diffuse midline glioma	4 (2.78%)
Other CNS tumors	20 (13.89%)
**Rare tumor**	72 (50.00%)
Rhabdomyosarcoma	17 (11.81%)
Ewing sarcoma	12 (8.33%)
Osteosarcoma	10 (6.94-%)
Other rare tumors	33 (22.92%)

### Feasibility

For the entire study cohort, the average number of days from biopsy to DNA/RNA sequencing was 10 days. The completed analysis and drug prediction report, comprising the possible drug targets identified through molecular data, was made available within an average of 17 days from biopsy. Tumor board decisions were made available within an average of 23 days from the biopsy date. Using the four‑drug combination agreed upon by the tumor board, therapy was initiated within an average of 38 days from biopsy.

The molecular tumor board was able to identify a precision medicine treatment plan for all patients. The drug choices were based on the tiered approach (Fig. 1C) with a discussion of DNA and RNA (*Z*-score > or < 2) findings and a literature review based on tumor type and pathways involved. The discussion involved possible drug combinations with the potential for inclusion of standard-of-care chemotherapy agents, safety of the combinations based on pharmacy input, and the ability to deliver the medication (intravenous versus oral depending on patient tolerability). In addition, the tumor board considered past therapies received by the patient, patient preferences for intensity of therapy, inpatient versus outpatient treatments, and central line access.

**Fig. 2:**
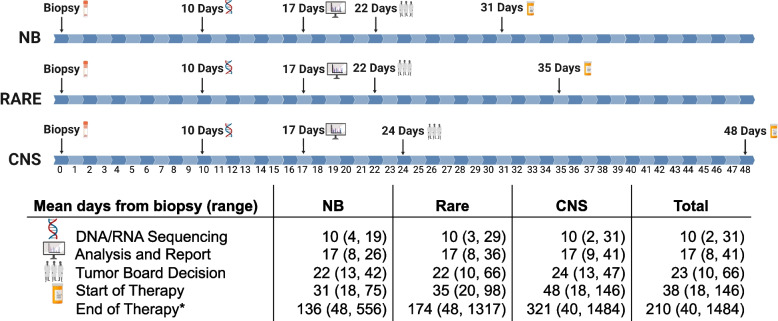
Feasibility was evaluated by a number of days from biopsy to DNA/RNA sequencing, analysis and report, tumor board decision, and the start of MGT. The average number of days from biopsy to these time points among the three tumor types is noted in the timeline. The ranges for the means are noted in the corresponding table. Values were calculated for all subjects who met the safety criteria (*n* = 144). For subjects still in therapy by the time of submission, November 1, 2019, was used as the end of therapy date, so as to not exclude these subjects from evaluation. *End of therapy is defined as the number of days from biopsy to the last dose of study drug administration

In stratifying the feasibility timeline by the three main pathologies, the average number of days (range) to DNA/RNA sequencing from the date of biopsy was 10 days (4,19) for neuroblastoma, 10 days (2,31) for CNS tumors, and 10 days (3, 29) for rare tumors (Fig. 2). The average number of days from biopsy to analysis and report was 17 days (8, 26) for neuroblastoma, 17 days (9, 41) for CNS tumors, and 17 days (8, 36) for rare tumors. On average, it took 22 days (13, 42) for neuroblastoma, 24 days (13, 47) for CNS tumors, and 22 days (10, 66) for rare tumors from the date of biopsy for a tumor board decision. The greatest variability in the feasibility timeline among the three tumor types was seen in the average number of days from the date of biopsy to the start of therapy, with 31 days (18, 75) for neuroblastoma, 48 days (18, 146) for CNS tumors, and 35 days (20, 98) for rare tumors.

It was found that 84.03% of subjects started all tumor board recommendations during cycle 1, and all but 4.86% were able to start all three or four drugs recommended (Table 2). Furthermore, 43.06% of subjects were able to start the entirety of the treatment plan on day 1 of cycle 1 and an additional 40.97% started during cycle 1. An additional 8.33% of subjects started all drugs during cycle 2, then 2.08% of subjects started all drugs during cycle 3, and 0.69% of subjects did not start all MGT agents until cycle 5. Overall, 95.14% of patients were able to start all medications recommended by the MTB.

**Table 2:** The feasibility of initiating a combination treatment regimen of up to four targeted agents is represented in the above table, with 84% of subjects able to start all recommended agents during cycle 1. All related and unexpected adverse events of grade 3 or higher were collected for this clinical trial and reported to the BCC. Only one grade 3 related and unexpected hematologic toxic effect occurred during this trial. Six grade 3 and one grade 4 non‑hematologic toxic effects that were related and unexpected occurred during the trial. Toxicity-associated events attributable to MGT causing a delay or reduction in treatment, such as medication holds, dose reductions, medication discontinuations, and cycle delays, were captured during cycles 1 and 2 via a retrospective roadmap review. Since this trial involved combination agents, these events were captured by the total number of events and the total number of subjects who experienced an event since subjects may have had > 1 drug held or reduced in dosage

NMTRC009 MGT feasibility and safety profile	*N* = 144		
**Started at least one tumor board recommended agent, no. of patients (%)**	144 (100%)		
**Started 100% of tumor board recommended agents, no. of patients (%)**			
Cycle 1	121 (84.03%)		
Cycle 2	12 (8.33%)		
Cycle 3	3 (2.08%)		
Cycle 5	1 (0.69%)		
Never	7 (4.86%)		
**Cycles, no. of patients (%)**			
1 cycle	19 (13.19%)		
2 cycles	45 (31.25%)		
3–10 cycles	57 (39.58%)		
> 10 cycles	23 (15.97%)		
	**Grade 3**	**Grade 4**	**Grade 5**
**Hematologic toxic effects (related and unexpected), no. of patients (%)**			
Anemia			
Lymphocytopenia			
Neutropenia			
Thrombocytopenia			
Leukopenia	1 (< 1%)		
**Non-hematologic toxic effects (related and unexpected), no. of patients (%)**			
Elevated ALT			
Elevated AST	1 (< 1%)	1 (< 1%)	
Dehydration	1 (< 1%)		
Infection	2 (< 2%)		
Oral mucositis	1 (< 1%)		
Pancreatitis	1 (< 1%)		
	**No. of events**	**No. of patients (%)**	
**Cycle 1 toxicity-associated events**			
Medication holds	90	45 (31.25%)	
Dose reductions	9	9 (6.25%)	
Medication discontinuations	9	8 (5.56%)	
Cycle delays	0	0 (0%)	
**Cycle 2 toxicity-associated events**			
Medication holds	105	52 (41.60%)	
Dose reductions	45	31 (24.80%)	
Medication discontinuations	21	15 (12.00%)	
Cycle delays	12	12 (9.60%)	

Time on the study was measured in days from the date of the initial biopsy to the date of the last administration of study therapy. For subjects with > 1 biopsies and > 1 MGT regimens, the off-therapy date from their last treatment was used. The average time (range) on the study for the entire subject cohort that met the safety criteria (*n* = 144) was 194 days (28, 1415). In stratifying by tumor type, the average time on study was 153 days (45, 556) for neuroblastoma subjects, 283 days (50, 1415) for brain tumor subjects, and 163 days (28, 1317) for rare tumor subjects. Forty-four of the 144 subjects (30.56%) who met the safety criteria were on study for at least 180 days. Eighteen of 144 (12.50%) subjects were on study for 365 days or more.

### Safety

Related and unexpected adverse events of grade 3 or higher are outlined in Table 2 for all subjects who met the safety criteria (*n* = 144). Each of these adverse events occurred in < 1% of the subject cohort, indicating nearly negligible levels of toxicity above expected for the drugs used during this trial. There was only one unexpected hematologic toxic effect that occurred during this trial, which was grade 3 leukopenia. There were seven occurrences of unexpected non‑hematologic toxic effects during this trial: grade 4 elevated ALT (1), grade 3 elevated AST (1), grade 3 dehydration (1), grade 3 infection (2), grade 3 oral mucositis (1), and grade 3 pancreatitis (1).

Forty-five subjects experienced a medication hold, 9 subjects experienced a dose reduction, and 8 subjects experienced a medication discontinuation during cycle 1. During cycle 2, 52 subjects had a medication hold, 31 subjects had a dose reduction, 15 subjects had a medication discontinuation, and 12 subjects experienced a cycle delay, due to expected and unexpected toxicities. No subject discontinued treatment due to toxicity.

### Efficacy

**Fig. 3:**
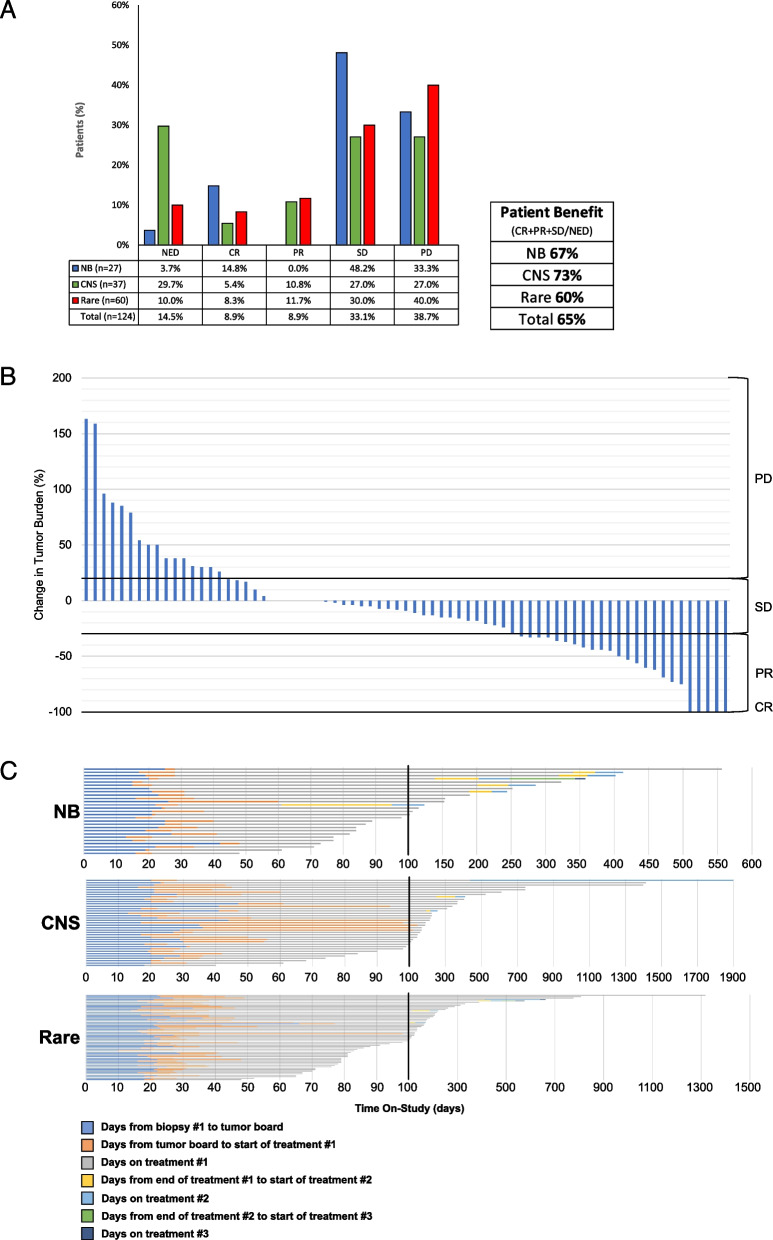
**A** For all subjects meeting the efficacy criteria (*n* = 124), best response rates were calculated using subjects’ best scan results while on‑study. Sixty-seven percent of neuroblastoma subjects, 73% of CNS tumor subjects, and 60% of other rare solid tumor subjects experienced stabilization of disease or better in this clinical trial. **B** Stable disease was defined as neither a sufficient decrease in tumor burden to qualify as PR nor a sufficient tumor burden increase to qualify as PD. Therefore, any change in tumor burden between ≤ 30% decrease and ≤ 20% increase all fell under SD. To better represent the change in tumor burden while on‑study, the percent change from pre‑cycle 1 scans to best scan while on‑study was determined for all subjects with measurable disease on imaging. A central review of all scans was performed by a single radiologist and measurements were performed using RECIST v1.1. Of the 73 subjects with measurable disease, 21 experienced an increase in tumor burden, 6 experienced no change, and 46 experienced a decrease in tumor burden. **C** Time on-study for all subjects meeting the safety criteria (*n* = 144) is represented in the swimmer plots. The axis scale breaks at day 100, marked by 2 dotted parallel lines. Each color represents a different time point of the trial (see color legend), accounting for multiple biopsied subjects

Of the 144 subjects, 124 met the efficacy criteria, including having at least one disease evaluation following the completion of at least 1 cycle of molecular‑guided therapy. Outcomes were measured by the subjects’ best scan while on the study. In total, 14.52% (18/124) remained NED status post-surgical resection, 8.87% (11/124) achieved CR, 8.87% (11/124) achieved partial response (PR), and 33.06% (41/124) maintained SD, while 39.71% (48/124) had progressive disease (Fig. 3A).

In stratifying by the three main tumor types, the response rates for neuroblastoma subjects were as follows: 3.57% (1/27) NED, 14.29% (4/27) CR, 46.43% (13/27) SD, and 33.33% (9/27) PD. Of the CNS tumor subjects, 29.73% (11/37) remained NED, 5.41% (2/37) achieved CR, 10.81% (4/37) achieved PR, 27.03% (10/37) remained stable, and 27.03% (10/37) progressed. The outcomes for subjects with rare solid tumors were 10.00% (6/60) NED, 8.33% (5/60) CR, 11.67% (7/60) PR, 30.00% (18/60) SD, and 40.00% (24/60) PD.

In stratifying based on genomic informed decisions, 0% of subjects were treated based on DNA alone, 19.5% were treated based on DNA and RNA clinical decision-making, and 80.5% of subjects’ clinical decision-making was based on RNA alone.

Patient benefit, calculated by the sum of the CR, PR, SD, and NED subjects divided by the sum of CR, PR, SD, NED, and PD subjects, was exhibited in 65% of all subjects, 67% of neuroblastoma subjects, 73% of CNS tumor subjects, and 60% of rare tumor subjects.

A central review of CT/MRI scans was performed for all subjects with measurable disease at C1D1. Change in tumor burden was calculated as a percent change between the pre-cycle 1 scans and the best scan while on-study. Of the 73 subjects with measurable disease, 63.01% had a decrease in tumor burden while 28.76% had an increase in tumor burden. Of the 33 subjects within the range of tumor burden change captured by SD (≤ 30% decrease and ≤ 20% increase), 22 had a decrease in tumor burden, 5 had an increase in tumor burden, and 6 had no change (Fig. 3B).

**Fig. 4:**
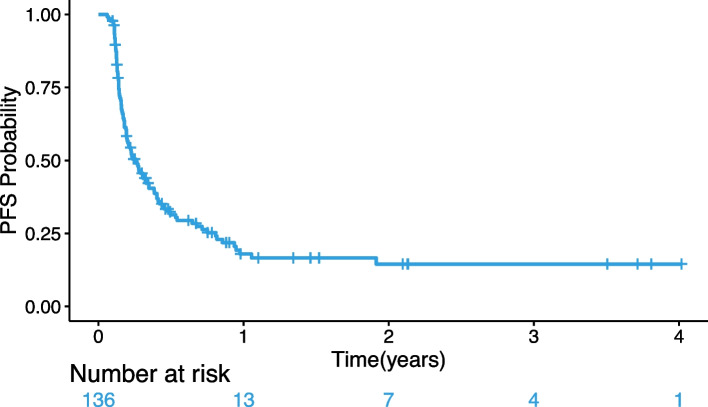
Kaplan–Meier plot of progression-free survival (PFS); the median progression-free survival (95% CI) for patients who received therapy was 0.26 years (0.19, 0.35)

The length of time on-study for all subjects meeting the safety criteria (*N* = 144) is shown in Fig. 3C. This plot presents a timeline for each subject on the study including the time between major study time points, including subsequent biopsies. Fourteen NB subjects, 27 CNS tumor subjects, and 32 rare tumor subjects were on study for > 100 days. Furthermore, 2 NB subjects, 7 CNS tumor subjects, and 5 rare tumor subjects remained in the study > 400 days. The median progression-free survival (PFS) time was 94.9 days with the PFS at 3 years being 15%; the Kaplan–Meier curve is shown in Fig. 4.

### Generation of cell line and PDX models

Cell lines and patient-derived xenograft models were established. Ninety-six unique cell lines were derived from subjects’ tumor biopsies (Additional file 1: Table S1). Of these 96, 26 were neuroblastoma, 23 were brain tumors, and 47 were rare tumors. Patient-derived xenograft models were successfully produced for a total of 47 subjects enrolled in this study, of which 15 were neuroblastoma, 1 was a brain tumor, and 31 were rare tumors.

### Molecular genomics

**Fig. 5:**
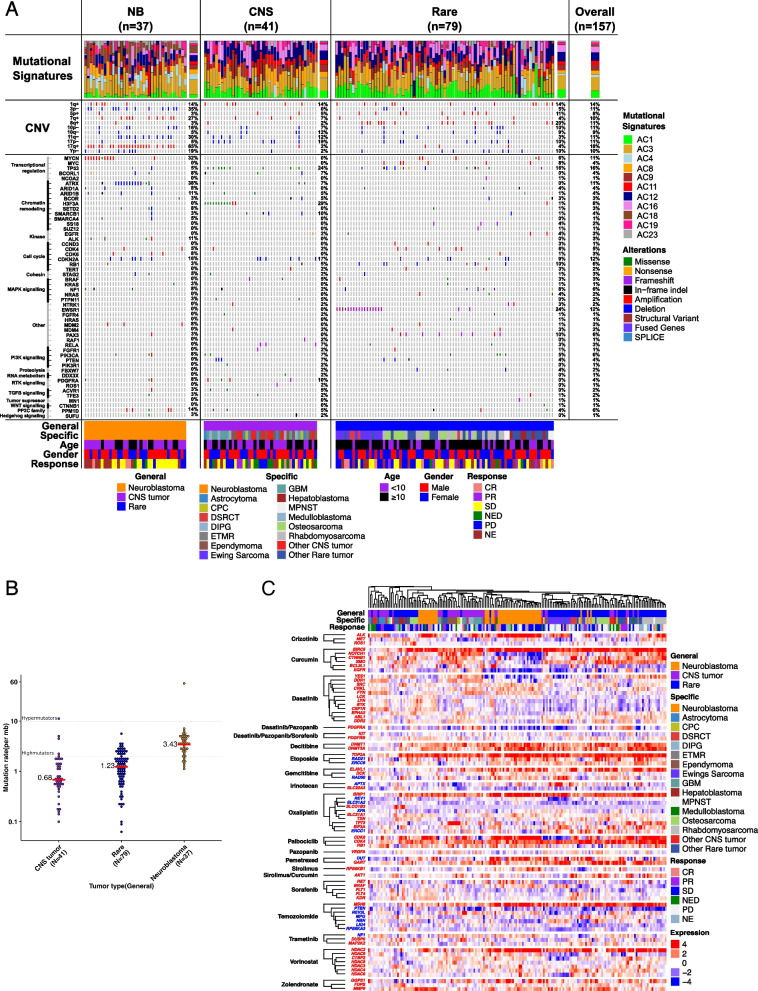
**A**, **B** Genomic landscape of relapsed and refractory childhood solid tumors. The genomic landscape of likely pathogenic driver mutations in 157 subjects with BCC with tumor and matched normal WES. **A** Mutational signatures are shown as a per-patient bar graph. Chromosome arm-level gains or losses, present in at least 15 subjects within the cohort, are displayed. Cancer genes bearing somatic SNVs, CNVs, SVs, and fusions are ordered according to general tumor type and frequency. **B** Somatic mutation burden is shown for each general tumor type. **C** Heatmap of molecular targets (over or under) of top sensitive drugs that are frequently expressed across MGT9 subjects. These drugs are selected based on number of times they are chosen across the subjects (> 10). Rows are divided by drugs, and genes are clustered to find unique or similar expression. Column clustering is performed using 78 genes to find the most similar subjects across different pathologies. Genes are colored with red (over) and blue (under)

In order to identify trends in genomic aberrations among the three diagnostic categories of NB, CNS, and rare tumors, mutational signatures, large-scale copy number variations (CNVs), and gene alterations were plotted into a comprehensive oncoprint (Fig. 5A, Additional file 1: Table S2). A total of 157 relapsed/refractory childhood tumor samples were analyzed and included (*n* = 37 NB, *n* = 41 CNS, and *n* = 79 rare) [31]. The median somatic TMB was 1.4 mutations per Mb (range 0.06–56.1; Additional file 1: Table S2). Mutation burden was highest in neuroblastoma (median 3.4) followed by rare tumors (median 1.2) and CNS tumors (median 0.7; Fig. 5B). Mutational signatures were plotted per individual tumor as well as averaged for each diagnostic category and the total cohort [40].

To characterize somatic copy number alterations, we evaluated large-scale and focal copy number alterations in the cohort. Somatic CNVs identified within neuroblastoma tumors included 17q + (78%), 7q + (43%), 1q + (30%), 2p + (8%), 11q‑ (43%), 3p‑ (43%), and 1p‑ (19%). The only CNV identified among CNS tumors was 17p‑ (27%). CNVs among rare tumors included 8q + (23%), 8p + (14%), 16q‑ (15%), and 17p‑ (8%). The CNS and rare tumor cohorts were substantially more heterogeneous, containing several more specific tumor types, which potentially explains the lower percent abundance of CNVs compared to neuroblastoma tumors.

Somatic gene alterations identified via whole-exome sequencing (WES) were plotted and vertically clustered by biochemical function, pathway relationship, and/or cell‑cycle control mechanism. WES of 37 NB tumors revealed MYCN amplification in 30%, ATRX deletion in 38%, TP53 alterations in 5%, and ALK mutations in 11%. Other genes of potential interest commonly altered in neuroblastoma tumors included cell cycle regulators (CDK4 [5%], CDK6 [8%], and CDKN2A [16%]), chromatic remodelers (ARID1A [8%], ARID1B [11%], and SETD2 [8%]), and other genes of unknown significance (BIRC6 [11%], MDM2 [8%], RNF213 [16%], and ERBB4 [11%]). The most common missense mutation among CNS tumors was in the H3F3A gene (29%). Furthermore, all CNS tumors possessing this mutation were considered HGGs (DIPG, GBM, and astrocytoma). Other common alterations of potential interest among CNS tumors included TP53 (24%), SMARCB1 (10%), CDKN2A (15%), and PDGFRA (12%). The canonical genetic alteration among rare childhood tumors is the EWSR1 fusion in Ewing sarcoma. WES of 79 rare tumors revealed the EWSR1 gene modified as a fusion, translocation, or nonsense mutation in 22% of the rare tumor cohort. The majority of rare tumor subjects that exhibited a mutated EWSR1 gene had a specific diagnosis of Ewing sarcoma (other diagnoses included desmoplastic small round cell tumor and other rare tumors). Other potential genes of interest commonly mutated among rare tumors included TP53 (16%), CDK2NA (10%), RB1 (10%), and LRP1B (14%).

### Gene expression signatures

To investigate the gene expression patterns in relapsed and refractory childhood solid tumors, a heatmap of molecular targets (genes) for the top selected drugs (chosen > 10 times) is shown in Fig. 5C. Unsupervised hierarchical clustering analysis of the 78 genes across the cohort of subjects with RNASeq data was performed (*n* = 184 which included longitudinal samples) (Additional file 1: Table S3). Of the targetable genes analyzed, the most commonly overexpressed genes in NB included BIRC5 (93%), TOP2A (89%), ALK (87%), BRIP1 (82%), HDAC2 (82%), CDK6 (80), and CDK4 (71%). The most commonly overexpressed genes in CNS tumors included TOP2A (67%), BIRC5 (59%), CDK4 (50%), BRIP1 (49%), NOTCH1 (41%), SMO (39%), and TP73 (39%). The most commonly overexpressed genes in rare cancers included BIRC5 (88%), TOP2A (85%), CDK4 (82%), BRIP1 (76%), MSH6 (66%), and HDAC2 (62%). Individual RNA gene expression levels were not found to correlate with response outcomes (ORR/PFS) (Additional file 1: Table S4).

### Tumor heterogeneity and evolution

**Fig. 6:**
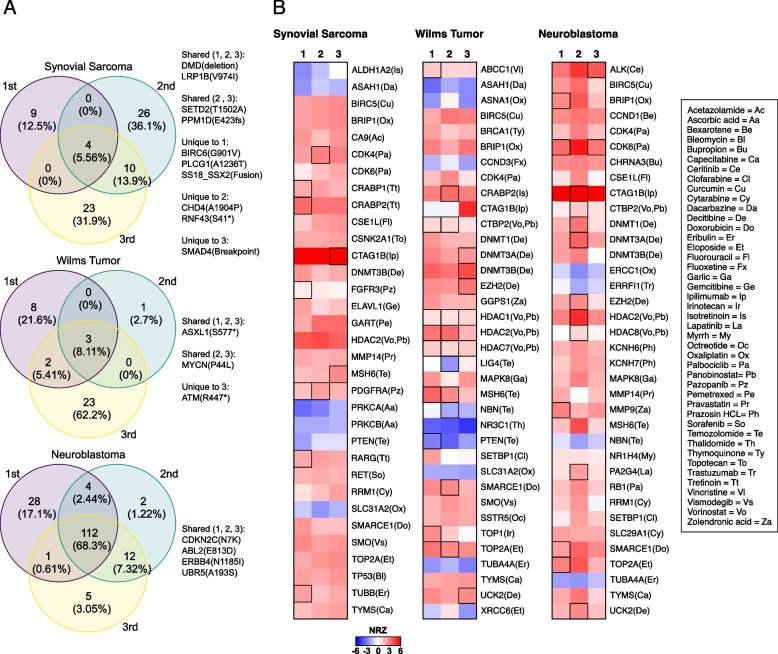
**A** Overlap of DNA events for three patients which each had three biopsies. The first was diagnosed with spindle cell synovial sarcoma and had 384 and 230 days between relapses. The second was diagnosed with Wilms tumor and had 412 and 88 days between relapses. The third was diagnosed with neuroblastoma and had 168 and 100 days between relapses. “1st” indicates the initial presentation while “2nd” and “3rd” indicate subsequent relapses. The number of overlapping mutations is shown with the percentage of overlapping mutations shown in parenthesis. Genes relevant to cancer are listed. **B** RNA *Z*-scores for three subjects each with three biopsies. Biopsy 1 is from the initial presentation, and biopsies 2–3 are from subsequent relapses. Selected genes are shown with the literature-associated drug target in parenthesis. Boxes are highlighted to indicate a molecular target of the drug chosen during the molecular tumor board

We next sought to investigate the genomic evolution of R/R childhood cancers by evaluating nine longitudinal tumor samples from three subjects (Additional file 1: Table S5). Tumor heterogeneity was evident in all subjects, and a majority of longitudinal samples demonstrated oncogenic events that are shared and unique across biopsies (Fig. 6A). In subject 1, a synovial sarcoma, LRP1B V974I hotspot mutation, was shared across all whereas SS18_SSX2 fusion was detected only in initial biopsy. In subject 2, a Wilms tumor; there were less oncogenic events, and ASXL1 S577* hotspot mutation was shared across all whereas MYCN P44L was present in later biopsies. In subject 3, a neuroblastoma; the number of protein-coding mutations was very high compared to other subjects. ERBB4 N1185I was present in all biopsies and was selected as a drug target (lapatinib). RNA expression differences of sensitive rule genes and their associated drugs were evident across three subjects among three longitudinal biopsies (Fig. 6B, Additional file 1: Table S6). The highlighted boxes indicate a molecular target of the drug chosen during the molecular tumor board.

## Discussion

This prospective, multicenter clinical trial tested the safety, feasibility, and efficacy of using tumor samples to obtain genomic data and generate a report used in a molecular tumor board for real‑time treatment decisions in subjects with relapsed/refractory childhood cancer. Most of the subjects enrolled in this study had received one or more lines of relapse treatment, prior to enrollment, further confirming the need for more effective individualized therapies for refractory childhood cancers.

The primary endpoint of this study was the feasibility of using genomic information in clinical decision-making, defined as days from biopsy to DNA/RNA sequencing, analysis and report generation, tumor board decision, and start of therapy. The average time to initiation of therapy from biopsy was within 38 days, a reasonable timeframe for treatment initiation. In comparing our feasibility timeline to previous precision medicine trials, we have demonstrated comparable turnaround times (TAT) for sequencing and treatment plans. Shukla et al. used both WGS and RNAseq and demonstrated a TAT of 9 days from biopsy to sequencing results, similar to our timeline of 10 days [43]. Mueller et al., although limited to newly diagnosed DIPG, also used WES and RNAseq and demonstrated a TAT of 21 days from biopsy to treatment plan, similar to our average of 23 days [44, 45]. This timeline suggests that the utilization of this process is a viable and feasible option for clinical decision-making and treatment of relapsed/refractory childhood cancers.

Given the paucity of randomized clinical trials by pharmaceutical companies in pediatric oncology, the majority of agents used in this study were considered off‑label, as is the case in most standard-of-care pediatric cancer treatment regimens. One factor leading to the variability in days from biopsy to the start of therapy was the substantial time needed to obtain authorization from insurance companies for the off‑label use of the drugs. In the case of CNS tumors, in which the average time was greater, recovery from surgery and/or radiation given post-surgery was also identified as factors contributing to delay in initiation of treatment.

The protocol for this trial mandated reporting of grade 3 or higher related and unexpected adverse events. The safety of this approach was evaluated in cycles 1 and 2. Although there was concern for potential increased toxicity when using combinations of targeted agents, the occurrence of reportable adverse events was extremely low. There was little unexpected associated toxicity for the MGT drugs used during this trial, supporting the safety of utilizing this method of therapy selection in the relapsed/refractory pediatric cancer population. Important aspects ensuring safety include the tumor board discussion, literature review and pharmacy review of drug interactions, and recommendations for dosing and schedule. Of note, the study captured unexpected grade 3 and 4 toxicities throughout the study and only evaluated treatment modifications occurring during the first two cycles. The chemotherapy regimens did result in expected toxicities for the medications as illustrated by drug interruptions and reductions as well as cycle delays (Table 2). These were felt to be manageable by the treating physicians. Treatment modifications were primarily attributed to expected medication toxicities. As such, drug modifications occurred based on known toxicity profiles. Medications were reduced only for those drugs attributed to have specific related toxicities (e.g., myelosuppression) and not all drugs in the treatment plan. No patient discontinued treatment due to toxicity.

Pediatric cancers historically possess a low mutation burden relative to adult cancers. The genomic landscape reported in this trial matches that previously reported [13, 20, 44, 46, 47]. This trial demonstrated that only a small proportion of relapsed/refractory pediatric cancer of patients would benefit from the sequencing of DNA alone. In fact, a common limitation among many previous precision medicine trials was that not all patients were found to have targetable genomic events that would warrant treatment [46]. This was due to a limited genomic workup such as gene panels or WES in the absence of RNAseq [13, 20, 26]. Another limitation of past studies is the use of single-agent therapy. The Zero Childhood Cancer Program precision medicine trial reported that 70% of molecular tumor board recommendations were single agents [20]. It has been well-established that multi-agent therapy is superior to single-agent therapy in overcoming resistance [16]. The addition of RNA transcriptome data facilitates understanding of activated pathways and provides added clinical utility in the pediatric population. As a result, all patients in our trial were offered a molecular treatment plan that included combination therapy of up to four drugs. Of note, DNA in combination with RNA data was used in 19.5% for MGT regimens whereas the remaining 80.5% of regimens were devised using RNA data alone, and there was not a statistically significant difference in outcomes for those treated based on DNA and RNA combination versus those treated using RNA alone. This further reinforces the importance of comprehensive genomic and transcriptomic analyses in pursuing targeted therapy [46].

The majority of mutational signatures have etiologies, features, and significance that have yet to be elucidated in the literature, but correlations can be identified between the presence of a specific signature and cancer types [40]. The mutational signature analysis revealed consistency with previous literature that identified an association between signature 18 and neuroblastoma. This signature was differentially expressed in neuroblastoma samples when compared to both CNS and other rare solid tumors. The etiology of this signature has been previously shown to be related to damage by reactive oxygen species [48], which is a long-known biological stimulus in NB [49].

This study confirms that clonal evolution in pediatric cancers does occur over time [30]. This change in tumor genomics with the evolution of new targets emphasizes the importance of re-biopsy and re-sequencing at each relapse. This approach may identify the different therapeutic approaches, as shown for patients in this study. The ideal frequency of biopsies and reassessments needs further exploration. In addition to tumor sequencing, the MAPPYACTS trial [50] correlated cfDNA from plasma suggesting that this may be an option for following tumor evolution over time without the need for biopsy.

## Conclusions

This trial demonstrated the feasibility, safety, and efficacy of a comprehensive sequencing model to guide personalized therapy for patients with any relapsed/refractory solid malignancy. Personalized therapy was well tolerated, and the response clinical benefit rate of 65% in these heavily pretreated populations suggests that this treatment strategy could be an effective option for relapsed and refractory pediatric cancers.

### Supplementary Information

**Additional file 1:** **Table S1.** Cell lines and mice models established. A total of 96 patients enrolled onto the NMTRC009 MGT trial had at least one tumor cell line generated in the laboratory setting. Since many subjects underwent multiple tumor biopsies and/or bone marrow biopsies, subjects may have >1 unique cell line, either from the same tumor obtained from different biopsy dates or from a different disease site (bone marrow). 47 subjects’ tumors underwent successful implantation into a NOD-SCID mouse to generate at least one PDX model. A total of 56 unique PDX models were generated.

## Data Availability

Sequencing data from this study has been deposited in the Database of Genotypes and Phenotypes (dbGaP) (31) under accession number phs002238.v1.p1 (https://www.ncbi.nlm.nih.gov/projects/gap/cgi-bin/study.cgi?study_id=phs002238.v1.p1).

## Abbreviations

AEs: Adverse events
BCC: Beat Childhood Cancer (formerly NMTRC)
CNS: Central nervous system
CTCAE: Common Terminology Criteria for Adverse Events
CR: Complete response
CNVs: Copy number variations
IRB: Institutional Review Board
ICH GCP: International Conference on Harmonization Guidance on Good Clinical Practice
MTB: Molecular tumor board
NB: Neuroblastoma
NMTRC: Neuroblastoma Medulloblastoma Translational Research Consortium
NED: No evidence of disease
PD: Progressive disease
PR: Partial response
POTRL: Pediatric Oncology Translational Research Laboratory at Penn State
RECIST: Response Evaluation Criteria in Solid Tumors
SNV: Single nucleotide variant
SD: Stable disease
WTS: Tumor whole-transcriptome sequencing
TAT: Turnaround times
WES: Whole-exome sequencing
